# Clinical evaluation of deep learning‐enhanced lymphoma pet imaging with accelerated acquisition

**DOI:** 10.1002/acm2.14390

**Published:** 2024-05-29

**Authors:** Xu Li, Boyang Pan, Congxia Chen, Dongyue Yan, Zhenglin Pan, Tao Feng, Hui Liu, Nan‐Jie Gong, Fugeng Liu

**Affiliations:** ^1^ Department of Nuclear Medicine, Beijing Hospital National Center of Gerontology; Institute of Geriatric Medicine, Chinese Academy of Medical Sciences Beijing People's Republic of China; ^2^ RadioDynamic Healthcare Shanghai People's Republic of China; ^3^ Laboratory for Intelligent Medical Imaging Tsinghua Cross‐strait Research Institute Beijing People's Republic of China; ^4^ Department of Engineering Physics Tsinghua University Beijing People's Republic of China

**Keywords:** deep learning, low‐dose imaging, lymphoma, PET

## Abstract

**Purpose:**

This study aims to evaluate the clinical performance of a deep learning (DL)‐enhanced two‐fold accelerated PET imaging method in patients with lymphoma.

**Methods:**

A total of 123 cases devoid of lymphoma underwent whole‐body 18F‐FDG‐PET/CT scans to facilitate the development of an advanced SAU2Net model, which combines the advantages of U2Net and attention mechanism. This model integrated inputs from simulated 1/2‐dose (0.07 mCi/kg) PET acquisition across multiple slices to generate an estimated standard dose (0.14 mCi/kg) PET scan. Additional 39 cases with confirmed lymphoma pathology were utilized to evaluate the model's clinical performance. Assessment criteria encompassed peak‐signal‐to‐noise ratio (PSNR), structural similarity index (SSIM), a 5‐point Likert scale rated by two experienced physicians, SUV features, image noise in the liver, and contrast‐to‐noise ratio (CNR). Diagnostic outcomes, including lesion numbers and Deauville score, were also compared.

**Results:**

Images enhanced by the proposed DL method exhibited superior image quality (*P* < 0.001) in comparison to low‐dose acquisition. Moreover, they illustrated equivalent image quality in terms of subjective image analysis and lesion maximum standardized uptake value (SUVmax) as compared to the standard acquisition method. A linear regression model with y = 1.017x + 0.110 ($R^{2}=\;1.00$) can be established between the enhanced scans and the standard acquisition for lesion SUVmax. With enhancement, increased signal‐to‐noise ratio (SNR), CNR, and reduced image noise were observed, surpassing those of the standard acquisition. DL‐enhanced PET images got diagnostic results essentially equavalent to standard PET images according to two experienced readers.

**Conclusion:**

The proposed DL method could facilitate a 50% reduction in PET imaging duration for lymphoma patients, while concurrently preserving image quality and diagnostic accuracy.

## 1 INTRODUCTION

Non‐Hodgkin lymphoma (NHL) is the thirteenth most common cancer in 2020, with Hodgkin lymphoma ranking twenty‐eighth, responsible for 2.8% and 0.4% of newly diagnosed cancer cases worldwide.^1^ Despite its prevalence, lymphoma is considered to be one of the most curable cancers in its early stages, emphasizing the significance of early‐stage detection.^2^, ^3^ 2‐deoxy‐2[18F]fluoro‐D‐glucose ([18F]F‐FDG) PET is a widely used nuclear medicine imaging modality that plays a major role in diagnosing, staging and restaging of various tumors, especially lymphoma.^4^, ^5^, ^6^ [18F]F‐FDG‐PET is performed repeatedly throughout the diagnostic and treatment process,^5^ with a standard dose of approximately 3.7‐5.55 MBq/kg (or 0.1‐0.15 mCi/kg) each time.^7^, ^8^ In light of the substantial frequency of ^18^F‐FDG‐PET examinations, an escalating apprehension has arisen concerning the prospective hazards associated with cumulative radiation exposure and its potential role in instigating carcinogenic processes.^9^ Therefore, the imperative for employing low‐dose PET is underscored to mitigate the radiation burden for lymphoma patients.

Numerous algorithms have been proposed to minimize the administered dose while maintaining image quality. These algorithms can be classified into two main groups: iterative reconstruction algorithms, such as OSEM^10^ and Bayesian penalized likelihood reconstruction^11^ and image post‐processing methods, like mapping‐based sparse representation,^12^ non‐local mean filter,^13^ hybrid spatial‐frequency domain filtering,^14^ context modeling approach,^15^ and multi‐resolution post‐processing method.^16^ However, most conventional approaches tend to reduce spatial resolution and quantitative accuracy, while also generating undesirable over‐smoothed structures.

In the past few years, various deep learning (DL) methods have been introduced for medical imaging tasks such as segmentation, lesion detection, and artifact reduction.^17^ DL‐aided PET imaging has been reported to restore standard dose‐like PET images from low‐dose data, such as 50%^18^ or 10%^19^ of the standard dose. Recently, the DL method based on the 3D U‐net was demonstrated can effectively reduce image noise and control bias, even for sub‐centimeter small lung nodules, when generating standard dose PET using 10% low count down‐sampled data.^20^ Zhou et al. also evaluated supervised CycleGAN model in terms of maximum standardized uptake value (SUVmax), and mean standardized uptake value (SUVmean).^21^

In this study, we apply a DL‐based image enhancement method to the most commonly seen lymphoma in different stages. We evaluate the quality of restored low‐dose PET from patients diagnosed with lymphoma, but handled by a DL model trained on a dataset without lymphoma cases. We also present a comprehensive and systematic clinical assessment approach for lymphoma cases to validate the effectiveness and reliability of DL based image augmentation method, including lesion number and the Deauville score for lymphoma. Deauville score currently is considered to be the international standard,which is a five‐point scale based on a visual comparison between the FDG uptake of lymphoma focus and the uptake of liver or mediastinal blood pool, respectively.^22^ Additionally, conventional metrics, such as image quality and SUV (standardized uptake value) measurements are also considered for a comprehensive evaluation.

## 2 METHOD

### 2.1 Patients data

Two cohorts comprising a combined total 162 participants were recruited for the purpose of this investigation. The first group of 123 subjects without lymphoma who underwent diagnostic [18F]F‐FDG‐PET/CT examinations between April 2020 and February 2022 were used as training datasets. This group included 75 males and 48 females, with a mean age of 64.8 ± 12.6 (range: 34−88) years. The second group consisted of 39 subjects (19 males, 20 females) with histologically confirmed lymphoma who had at least one lesion with high FDG uptake and underwent PET/CT examinations between February 2021 and 2022. These subjects with a mean age of 59.5 ± 16.6 (range: 26−85) years were either in the pre‐treatment phase (*n* = 11) or undergoing treatment (*n* = 28). There was diffuse large B‐cell lymphoma (DLBCL, *n* = 21), follicular cell lymphoma (FL, *n* = 6), Hodgkin lymphoma (HL, *n* = 6), mucosa‐associated lymphoid tissue lymphoma (MALT, *n* = 3), Burkitt lymphoma (*n* = 1), plasmablastic lymphoma (PBL, *n* = 1), and T‐cell lymphoma (*n* = 1). According to the Ann Arbor staging system, 10 (25.6%) subjects were at stage II, 11 (28.2%) at stage III, and 18 (46.2%) at stage IV.

### 2.2 Imaging protocol

All PET/CT examinations were performed using Vereos PET/CT, Philips, employing a list‐mode scanning protocol. The [18F]F‐FDG used in this study was produced and provided by Beijing Atom High Tech Co., Ltd., with a radiochemical purity over 95%. Each participant underwent a minimum 4‐h fasting period and was required to have a controlled blood glucose level <11.1 mmol/L prior to receiving an intravenous injection of 5.18 MBq/kg (0.14 mCi/kg) [18F]F‐FDG. After a 60‐min period of rest, a spiral CT scan in a supine position from the base of the skull to the proximal thighs was conducted with a tube voltage of 120 kV and flexible tube current adjusted by CARE dose technology. Subsequently, a PET scan (1 min/bed, 5−8 beds) was conducted in the three‐dimensional mode with the same coverage. The list‐mode data were reconstructed with an ordered subset expectation‐maximization algorithm (two iterations, eight subsets) with attention correction and a time‐of‐flight model followed by a Gaussian filter. The first half of the list‐mode data was reconstructed under the identical protocol in order to generate the 1/2 low‐dose PET.

### 2.3 Network architecture

**FIGURE 1:**
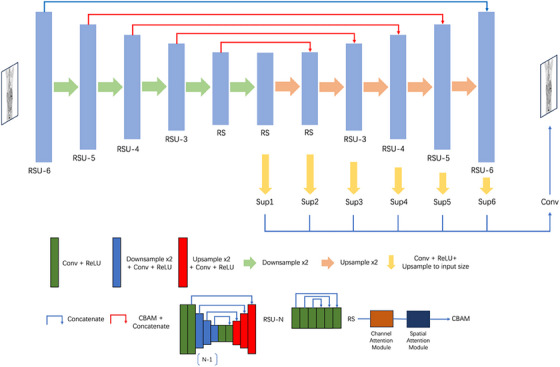
Architecture of proposed SAU2Net. The network backbone is a U2Net model with batch normalization layers removed. Adapted CBAM blocks are incorporated within the long skip connections.

**FIGURE 2:**
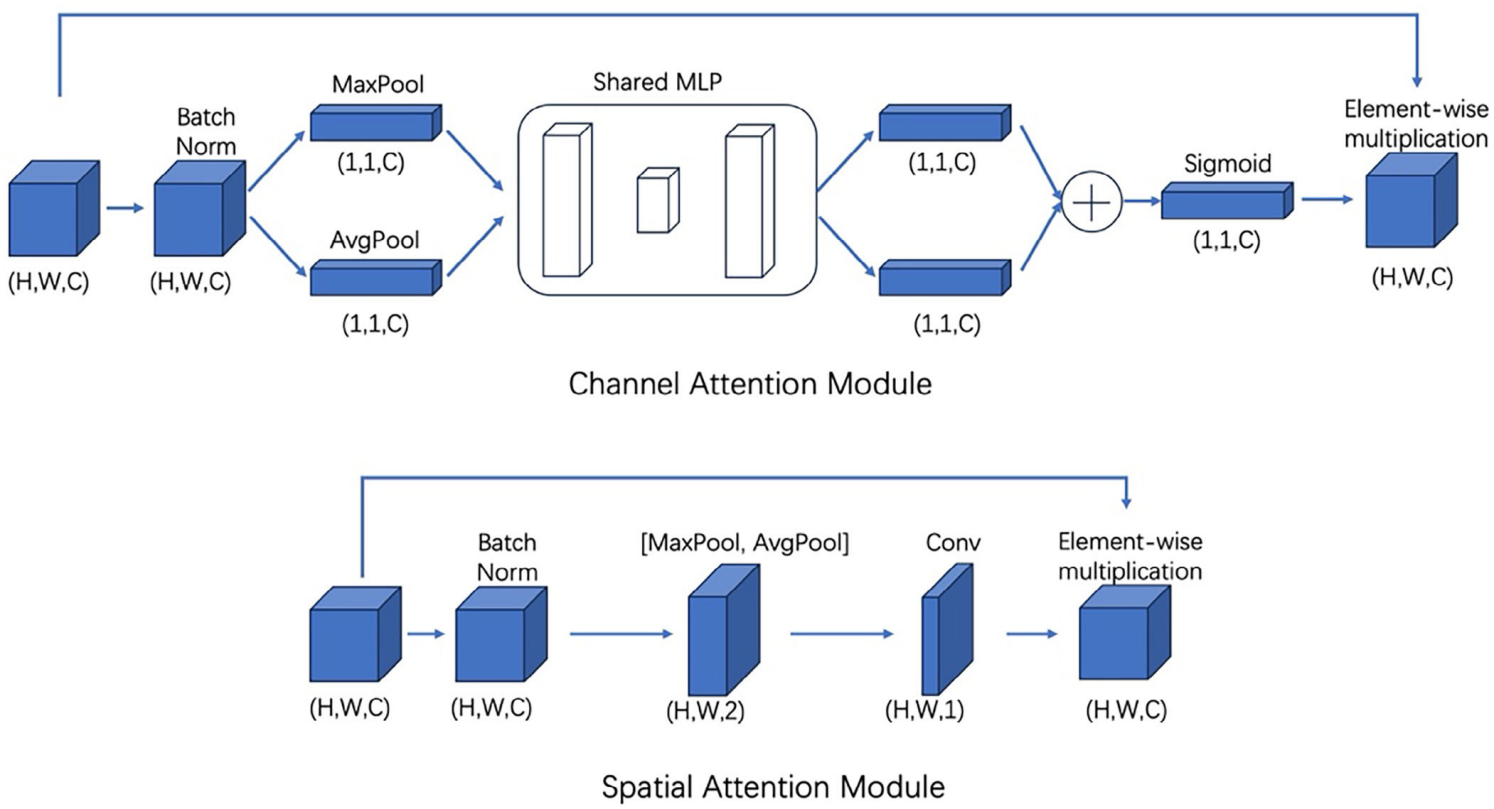
The adapted CBAM module consists of a channel attention module and a spatial attention module. A batch normalization layer is introduced before pooling layers to adapt the data distribution of PET images represented in SUVs.

Inspired by the refined segmentation results achieved by U2Net and the success of attention mechanism, we propose a novel framework named Self‐Attention U2Net (SAU2Net) for PET image enhancement. The structure of the network is depicted in Figures 1 and 2.

The backbone of the network consists of a U2Net^23^ adjusted to accommodate the specific requirements of image generation tasks. U2Net is a two‐level nested U‐structure initially designed for single‐image salient object detection. To modify the model for image generation tasks, batch normalization layers are removed from the original design. Multiple slice input substitutes for single slice input to fully leverage the adjacent spatial information of PET slices. We also propose the incorporation of the convolution block attention module (CBAM^24^) within the skip connection component of each stage in the U2Net architecture. Each CBAM block consists of a channel attention module and a spatial attention module (Figure 2). Batch normalization layers are incorporated prior to pooling layers while generated attention map multiplies the original input to adapt the data distribution of PET images and facilitate the model convergence.

### 2.4 Training details

For each case, the first and the last 20 slices were excluded due to the presence of strong noise in the images. Three contiguous slices were cropped into the size of 3 × 256 × 256 using the center part of the images before being fed into the network. The network was initialized using Xavier.^25^ Deep supervision was used as directed by.^23^ Loss function of each stage was implemented as follows to optimize image generation.

$$l\;=l_{pixel}\;+\gamma l_{perceptual}$$

where $l_{pixel}$ represent pixel wised loss and $l_{perceptual}$ stands for perceptual loss. Adam optimization was used to train the network, where initial learning rate was set to 0.004, and the learning rate dropped by half every 10 epochs. Other parameters were set to their default values (betas = (0.9, 0.999), eps = 1e‐8, weight decay = 0). The network was trained for 200 epochs.

### 2.5 Objective image analysis

#### 2.5.1 Quantitative analysis

Two computational metrics, namely Peak‐signal noise ratio (PSNR) and structural similarity (SSIM)^26^ were used to quantitatively measure the performance of the networks.

***PSNR***: The ratio of the maximum power of an original image to the noise power of the distorted image, which is formulated as:

$$PSNR\;=\;10\frac{MAX^{2}}{MSE}\;dB$$

where *MAX* is the peak intensity of the standard‐dose image, and MSE is the absolute mean square error between standard‐dose image and low‐dose image.

***SSIM***: computed perceived image quality of an image which is measured between noise image (low‐dose PET images) and reference image (standard dose PET images):

$$SSIM\;=\frac{\left(2\;\mu_{m}\mu_{n}+c_{1}\right)\left(2\sigma_{mn}+c_{2}\right)}{\left(\mu_{m}^{2}+\mu_{n}^{2}+c_{1}\right)\left(\sigma_{m}^{2}+\sigma_{n}^{2}+c_{2}\right)}\;$$

*µ*_m_, *µ*_n_, *σ*_m_, *σ*_m_ are mean and standard deviation in the patch centered at pixel (i, j), *σ*_mn_ is cross‐correlation between *m* and *n*, **c_1_** and **c_2_** are constants used to avoid a zero division.

#### 2.5.2 SUV analysis

The SUV serves as a widely employed metric for assessing PET uptake, playing a crucial role in clinical diagnosis.^27^ Lesions and liver were designated as regions of interests (ROIs) for SUV calculation. Initial delineation of ROIs was performed on standard‐dose PET images and subsequently transferred to low‐dose PET images and enhanced PET images. SUVmax, SUVmean and standard deviation (SD) were calculated for further analysis.

Bland‐Altman analysis was utilized to evaluate the agreement between different scan protocols.^28^ Moreover, we calculated the signal‐to‐noise ratio (SNR) of liver and contrast‐to‐noise ratio (CNR) of lesions with the highest metabolic activity,^29^ which is formulated as:

$$SNR\;=\frac{\;\frac{1}{N}\sum_{i=1}^{N}SUV_{mean}^{i}\left(lesion\right)}{SD\left(liver\right)}\;$$

$$CNR\;=\frac{SUV_{mean}\left(lesion\right)-SUV_{mean}\left(liver\right)}{SD\left(liver\right)}\;$$

### 2.6 Subjective image analysis

Two nuclear medicine physicians, with 10 and 15 years of experience in PET reading, independently evaluated the images in a randomized manner. The image quality was evaluated using the 5‐point Likert scoring system with the guideline shown in Table 1. Diagnostic evaluation was based on the number of lesions and the Deauville score. If more than 10 lesions can be found, the lesion number is set to 10. The physicians were unaware of the specific technique employed and had the freedom to review all the axial, sagittal, and coronal image series during the evaluation process. If a consensus could not be reached, a third physician will be consulted.

**TABLE 1:** 5‐point Likert scale for image quality, lesion conspicuity, and image sharpness.

Score	Overall image quality	Lesion conspicuity	Image sharpness
1	Unacceptable image quality and nondiagnostic	Invisible and extremely difficult to identify	Significant loss of image detail, nearly impossible to discern any details
2	Suboptimal image quality with impairment of diagnostic confidence	Difficult to identify but some details are recognizable	Partial loss of image detail, difficult to discern details
3	Acceptable image quality and not affecting the diagnostic confidence	Moderate clarity, with most details identifiable	The image has moderate clarity and sharpness, with most details discernible
4	Good image quality and diagnostic confidence	Clearly visible, with details easily recognizable	The image details are clear and sharp, with high clarity
5	Excellent image quality and absolute diagnostic confidence	Extremely clear, with all details very easily identifiable	The image is extremely clear, with very high detail sharpness and no blurriness

## 3 RESULTS

### 3.1 PSNR SSIM

PSNR and SSIM were used to validate the performance of our network in enhancing image quality. Comparisons are presented between the standard dose group and the low‐dose group, denoted as **Low**. The low‐dose images are enhanced by the proposed DL approach, which we denote as **DL**. Kernel Density Estimation (KDE) and histogram techniques are employed to visualize the distributions.

**FIGURE 3:**
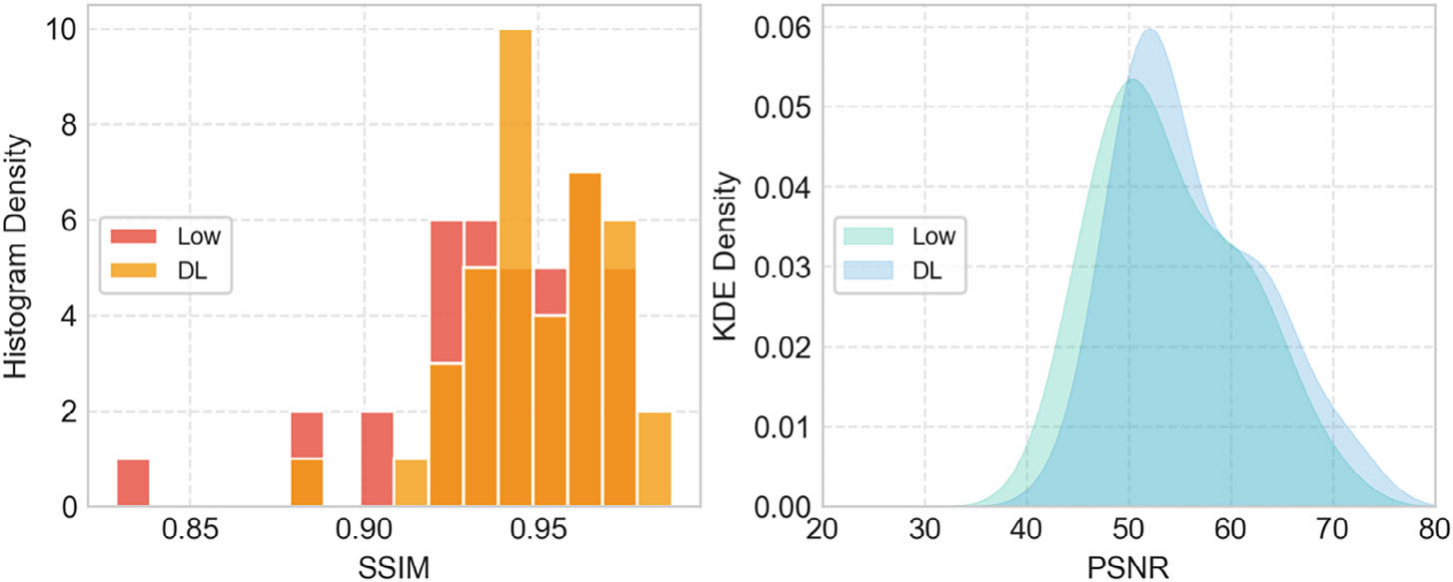
SSIM (histogram) and PSNR (KDE curves) with enhancement method proposed. Low, DL represent low‐dose—standard dose experiment group and deep learning enhanced low‐dose—standard dose group correspondingly.

Shown in Figure 3, KDE curves demonstrate the distributions of PSNR. The histogram of **DL**, represented by a blue line with shading, demonstrates a higher PSNR value compared to its counterpart, suggesting an overall improvement of image quality. Resembling conclusions can also be supported by SSIM statistics. After improvement, there are fewer cases with poor SSIM observed from the shorter left tail bins in **DL** compared to **Low**. A paired‐sample t‐test revealed a significant difference between the **Low** and **DL** groups in terms of both PSNR and SSIM.

### 3.2 SUV performance

**FIGURE 4:**
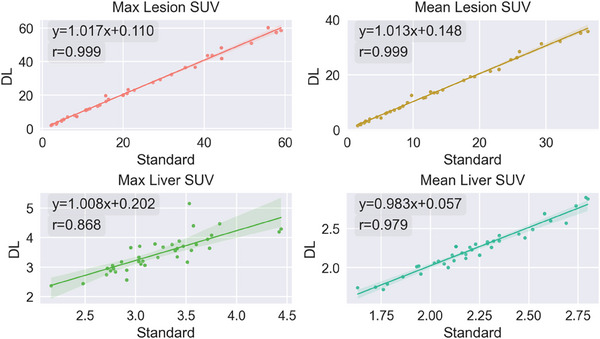
Fitted linear regressions and Pearson correlations coefficient for lesion SUV and liver SUV. Where x represents SUV value from the standard acquisition and y stands for SUV value from DL‐enhanced image. The shaded regions surrounding the lines indicate the regions of 99% confidence level SD Liver SUV: Standard deviation of counts within hepatic region which is considered as benchmark level.

Linear regression and Pearson correlation coefficient (*R* ^2^) were utilized to assess the SUV level after processing. Remarkably strong correlations revealed by *R* ^2^ of 1.0, as depicted in Figure 4, indicated that the enhanced PET scans exhibit a highly identical count distribution with that of standard‐dose images. Intercepts fall within the range of [−0.078, 0.202] for all SUV.

**FIGURE 5:**
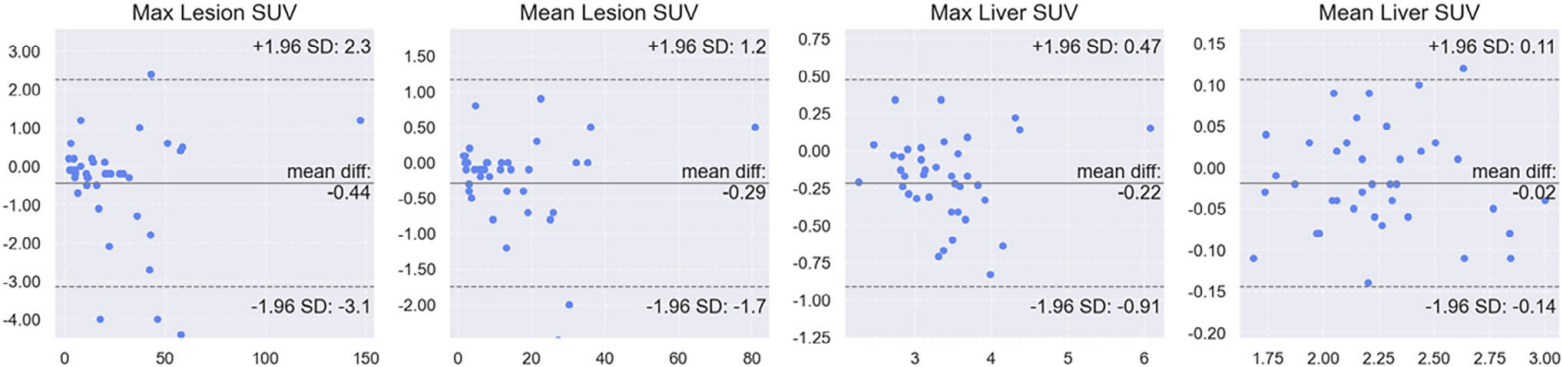
Bland‐Altman analysis of SUV for lesion and liver. Where solid line in the middle and two dashed lines aside are mean difference and ± 1.96 standard deviations, region with two dashed line is with 95% limits of agreement (LOA). x and y axis are mean value and difference value each.

A Bland‐Altman analysis, shown in Figure 5, evaluated SUV measurements. The PET data points displayed a dispersion pattern around the mean difference line, with the majority falling within the predefined 95% limits of agreement (LOA), indicating high agreement. However, a few outliers were observed in lesion SUVmax and liver SUVmean, exceeding the 95% LOA. Overall, the analysis demonstrates robust agreement between the enhanced images from the proposed method and the standard‐dose images in terms of SUV characteristics.

**FIGURE 6:**
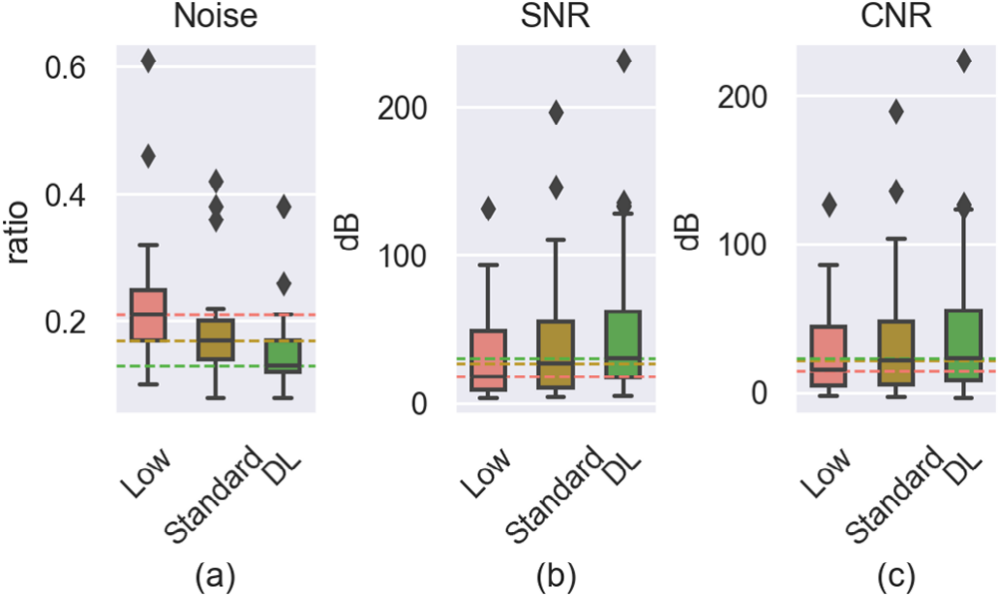
Boxplot of Noise, SNR and CNR on low‐dose images (Low), standard‐dose images (Standard) and enhanced images (DL) apiece.

The boxplot of noise, SNR and CNR on low‐dose PET, standard‐dose PET and DL‐enhanced low‐dose PET images is shown in Figure 6. The noise level of images enhanced from low‐dose data was significantly reduced compared to the original scans and even was lower than the noise level of standard‐dose images. The evaluation of SNR and CNR also supported the improvement of DL group compared to the low scan. Enhanced low‐dose images remarkably exhibited superior performance compared to standard‐dose images, as evidenced by higher median, first quartile, and third quartile in both SNR and CNR. All three plots consistently demonstrated that the proposed method effectively mitigated the noise level associated with low‐dose data, resulting in improved image quality and enhanced consistency. The quantitative consistency analysis in Table 2 also proved the result.

**TABLE 2:** Subjective and objective image analysis statistics with.

Variable	Group low recipients number (*n* = 39) Mean ± SD	Group standard recipients number (*n* = 39) Mean ± SD	Group DL recipients number (*n* = 39) Mean ± SD	*P‐*value (L‐DL)/(S‐DL)
**Subjective image analysis variables**				
Overall image quality	2.385 ± 0.702	3.077 ± 0.572	3.282 ± 0.552	.001^*^ /022^*^
Lesion conspicuity	3.000 ± 0.847	3.333 ± 0.523	3.385 ± 0.582	.001^*^ /0.323^*^
Image sharpness	2.462 ± 0.674	3.103 ± 0.590	3.282 ± 0.552	.001^*^ /.018^*^
Number of lesions	7.718 ± 3.587	7.692 ± 3.624	7.744 ± 3.557	1.00^†^ /1.00^†^
Deauville score	4.641 ± 0.832	4.615 ± 0.836	4.667 ± 0.857	1.00^†^ /1.00^†^
**Objective image analysis variables**				
Lesion SUVmax	26.056 ± 26.222	25.649 ± 26.045	25.205 ± 26.027	.001^†^ /.545^†^
Lesion SUVmean	14.928 ± 14.793	14.846 ± 14.642	14.556 ± 14.633	.001^†^ /.021^†^
Liver SUVmax	4.074 ± 1.252	3.527 ± 0.708	3.309 ± 0.654	.001^*^ /.072^*^
Liver SUVmean	2.244 ± 0.313	2.256 ± 0.315	2.237 ± 0.312	.849^*^ /.001^*^
**Image quality variables**				
Noise	0.222 ± 0.090	0.178 ± 0.070	0.149 ± 0.054	001^*^ /.001^*^
SNR	31.703 ± 29.370	42.528 ± 43.307	48.469 ± 48.127	.001^*^ /.010^*^
CNR	26.795 ± 28.953	36.179 ± 42.609	41.073 ± 47.625	.001^*^ /.005^*^

^*^Paired. Sample *t‐*test (2‐tailed). *P *< 0.05 with a 95% CI was considered statistically significant.

^†^Wilcoxon matched‐pairs signed rank test. *P *< 0.05 was considered statistically significant.

Experimental Group Setting:

Group **Low**: Low ^18^F‐FDG dosage.

Group **Standard**: Standard ^18^F‐FDG dosage.

Group **DL**: Low ^18^F‐FDG dosage with enhanced image quality by our deep learning method.

### 3.3 Subjective image analysis

Thirty‐nine test cases including 117 images were engaged in the subjective investigations. For the purpose of simplicity, we made Group **Low** denote the low‐dose [18F]F‐FDG‐PET images, Group **Standard** denote the standard dose [18F]F‐FDG‐PET images and Group **DL** denote enhanced low‐dose images.

This single‐blinded investigation revealed a significant impact of the proposed method on image quality from many aspects. As a result, compared to Group Low, significant improvement can be observed in terms of overall image quality, Lesion conspicuity, Image sharpness, noise level, SNR and CNR from Group DL. Following enhancement, approximately 76.92% of the samples were deemed to have experienced an improvement in image quality according to the clinicians. Among these samples, approximately 64.10% displayed an increase of one level in their scores, while 12.82% exhibited a two‐level improvement. In addition, 46.15% of the samples initially rated as “poor” (score of 2) were elevated to a “fair” rating (score of 3), while 17.95% improved from “fair” to “good” (score of 4). Furthermore, a significant improvement was observed in the diagnostic capability of the images, with 5.13% transitioning from a “nondiagnostic” rating to a remarkable 7.69% advancement from “poor” to “good”. Importantly, no images were deemed to have deteriorated after enhancement.

And enhanced images achieved competitive lesion conspicuity to that of standard scan. Impressively, DL‐enhanced images got a better performance aspect of overall image quality, image sharpness, noise, SNR and CNR from physicians’ perspective. providing a solid foundation for the mean‐level changes. Put simply, our proposed method effectively elevated low‐dose images to a comparable level as the standard‐dose images, as indicated by the experts’ responses.

In terms of diagnostic criteria, namely lesion number, and Deauville score, no significant difference was found between the three groups. This result suggested twice the scanning speed will not mislead clinicians during the diagnosis of lymphoma. Additionally, there is a low risk of erroneously concluding the presence of a difference when no actual difference exists, suggesting a convincing changing after the processing, particularly in relation to liver SUVmax.

### 3.4 Special case analysis

**FIGURE 7:**
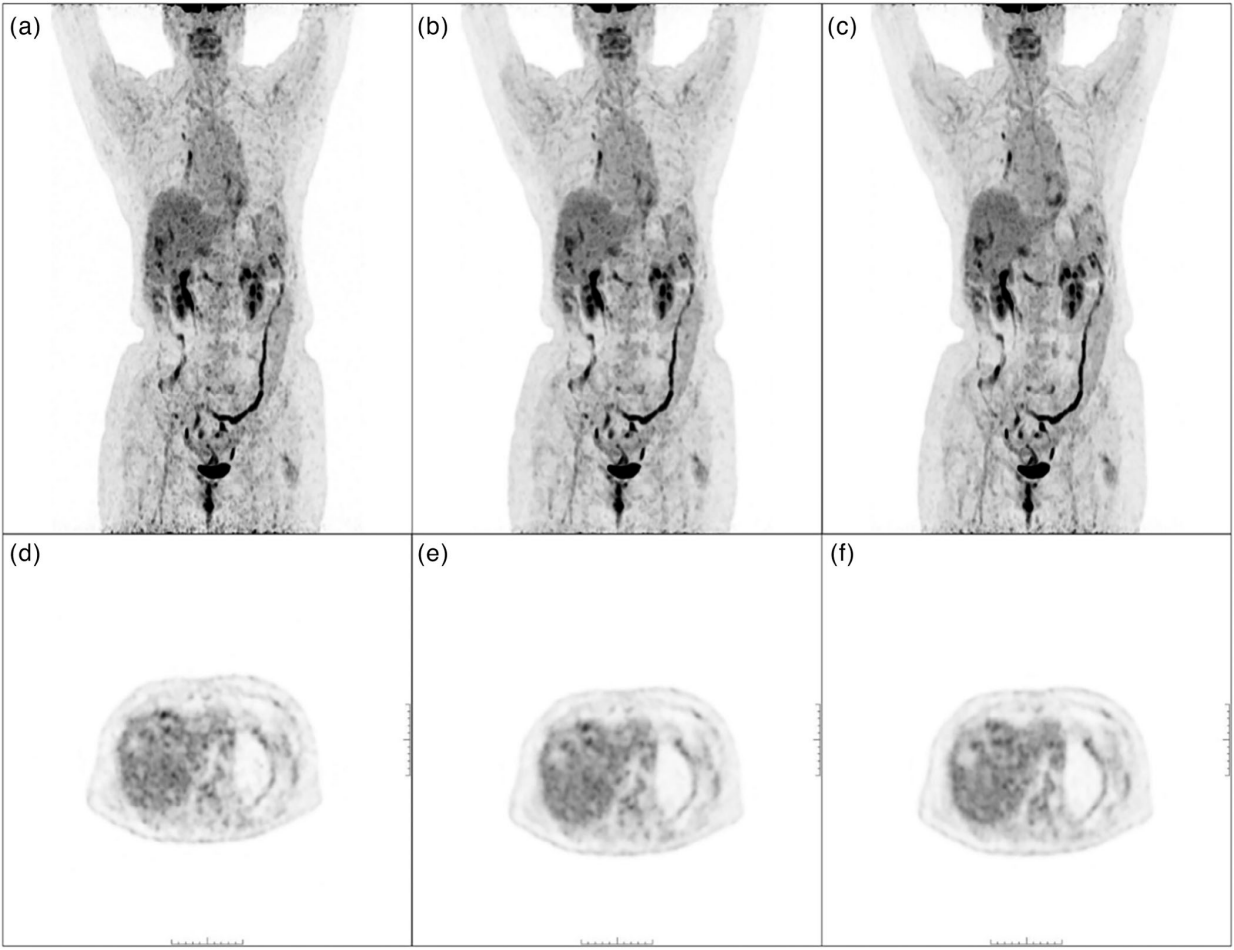
Visualization of low‐dose images (a, d), restored images that were generated using our method on the low‐dose images (b, e) and standard‐dose images (c, f) containing maximum intensity projection (MIP) and hepatic region. The restoration process effectively elevated the visual quality of the low‐dose image to a level that was visually comparable to the standard‐dose image.

**FIGURE 8:**
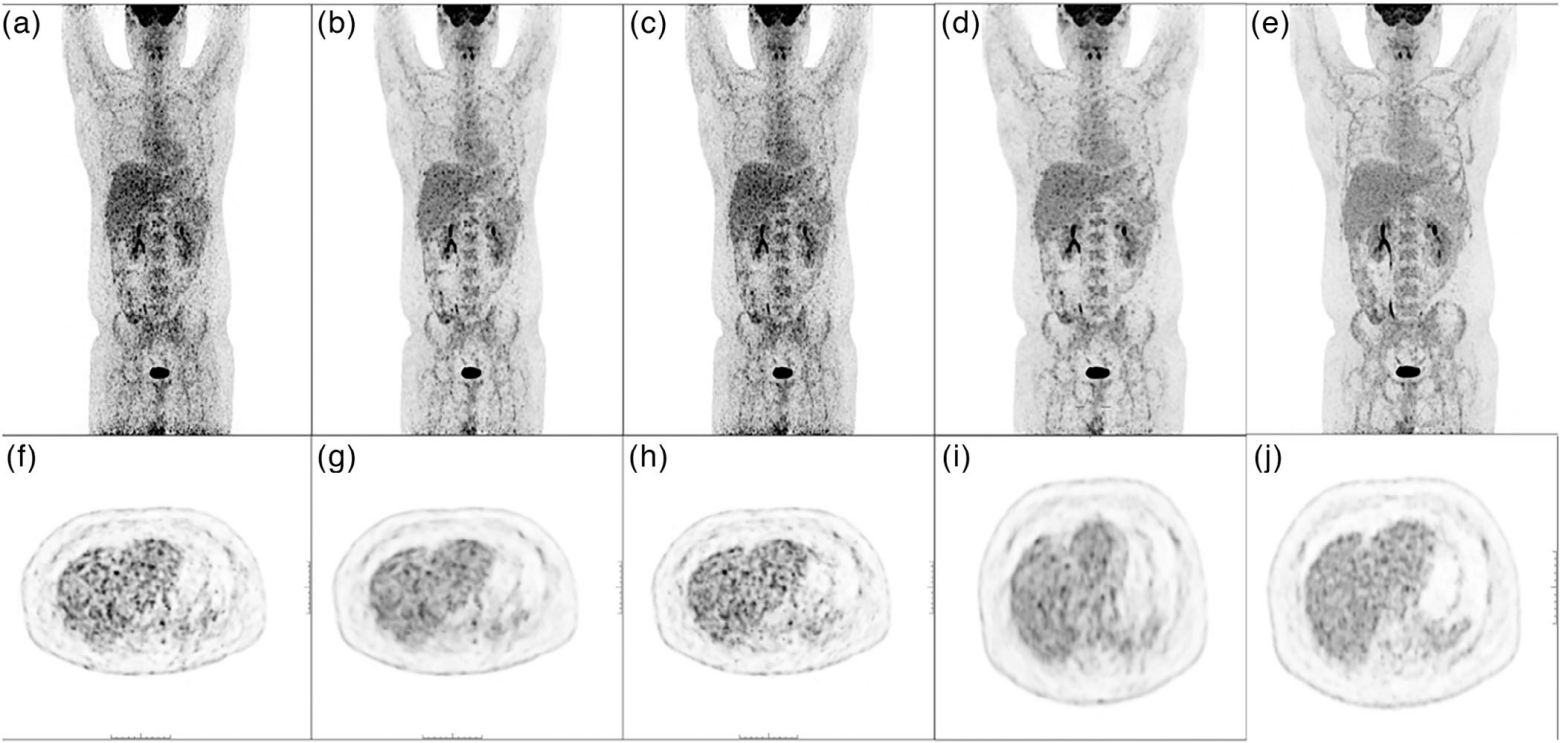
Visualization of low‐dose images (a, f), low‐dose restored images (b, g), standard‐dose images (c, h), standard‐dose restored images (d, i) and time‐added images (e, j) containing maximum intensity projection (MIP) and hepatic region. The restored process effectively elevated the visual quality of the low‐dose images to a level that was visually beyond the standard‐dose images, but still worse than that of the time‐added images. The restored process elevated the visual quality of the standard‐dose images to a level that visually comparable to the time‐added images.

Figure 7 showed the visual comparison of low‐dose PET, DL‐enhanced PET and the standard acquisition of a common case. In the source of our study, we found one severely obese male patient who weighed 115 kilograms. His restored images generated using our method on the low‐dose images effectively improved the visual quality of the low‐dose images to a level that surpassed even the standard‐dose images visually. But the visual quality of the restored images and the standard‐dose images did not meet the requirements for a diagnostic image, so this subject was excluded from the study. This patient underwent a repeat examination with added time to 1.5 min/bed on the same day and obtained a diagnostic image. Using our method, we restored the visual quality of the standard‐dose images again, and got enhanced images that were visually comparable to the time‐added images in Figure 8. This may indicate that even if severe obese patients need to be scanned for longer periods or injected with higher doses of radiopharmaceuticals than the average patient, as is well known, our method can also improve the image quality of the routine or standard dose. It is beneficial for severe obese patients to reduce the examination duration and radiation dose as much as possible.

## 4 DISCUSSION

DL algorithms could potentially provide a solution to the growing number of PET/CT examinations. Improving efficiency is beneficial as it can reduce the cost of PET and increase patient throughput for a single scanner. It may also be beneficial for patients who are prone to motion artifacts and cannot tolerate longer examinations. The use of lower doses may reduce the probability of secondary malignant tumors in patients underwent PET/CT examinations, especially for radiation‐sensitive populations.^30^, ^31^ With the aid of DL based image enhancement method, researchers intend to develop technique that can maintain the image quality and diagnostic efficiency while reducing the radiation dose or examination duration for patients undergoing PET/CT examination. This is particularly important for the lymphoma patients who repetitive PET/CT scans throughout the entire diagnosis and treatment process, and sometimes even for review purpose.

This study evaluated the performance of the proposed DL model on low‐dose PET images with 1/2 scan time. We evaluated the image quality using objective metrics, including SNR in the liver, CNR in the lesions, noise in the liver, SUVmax in the lesions and liver. We also assessed subjective metrics, such as lesion conspicuity and image sharpness. In addition, we evaluated the diagnostic accuracy by counting the number of lesions, and using the Deauville score through a single‐blind test conducted by two experienced physicians. In most cases, our proposed method effectively improved the low‐dose images to a comparable level as the standard‐dose images, and in some cases, even better. There was a strong agreement between the two groups of images in terms of SUV characteristics and Deauville score. This result agreed with the conclusions of previous studies. Wang et al.^32^ built a DL‐based reconstruction workflow using 1/3 list‐mode data and achieved comparable image quality to that of OSEM reconstructed images using full list‐mode data. Xing et al.^33^ reported superior performance regarding image noise and a reduction of image contrast in PET images reconstructed using a DL algorithm. In our research, lower noise in DL‐enhanced PET images was detected, but the reduction of image contrast was not observed.

The accuracy of the SUV is always a concern for DL‐based PET image enhancement. Weyts et al.^34^ found a significant decrease in SUVmax and SUVpeak for AI‐enhanced 45s PET compared to the standard 90s acquisition. Zhao et al.^35^ evaluated the SUV bias for lesions in different sizes and different dose levels. The best average SUVmax biases for all lesions are −3.7 ± 16.2% for 10% dose level and −5.2 ± 6.8% for 30% dose level. Our results showed a better average lesion SUVmax bias of −1.1 ± 6.2% and an average absolute SUVmax bias of 4.6 ± 5.2% for DL‐enhanced PET compared with 3.4 ± 9.7% and 6.2 ± 8.3% for low‐dose acquisition. The Wilcoxon matched‐pairs signed rank test showed no significant difference between DL‐enhanced PET and standard PET for lesion SUVmax and liver SUVmax.

One contribution of this work is that we trained the model using cases without lymphoma, but evaluated the model on a dataset with lymphoma. The objective and subjective results confirm the effectiveness of this strategy. No diagnostic deviation was discovered by the physicians for different types of lymphoma, including DLBCL, FL, HL and other commonly seen clinical types based on the criteria of lesion numbers, Deauville score, and SUV. This result hinted that the reliability of the DL method does not depend on whether certain types of data are included in the training dataset, but rather relied more on the features of the signals and the noise. From this point, this method could be applied to other cancers like breast cancer or melanoma.

Above experiments have shown an overall promising effect of our methods, there were still, however, some other issues that can be further discussed. Regression Plots, Bland‐Altman analysis and noise plots contributed collectively to the existence of outliers, spreading across quantitative metrics. Note that the abnormal cases were not contributed all by one patient. Instead of all, each patient might only contribute extreme values to some indicators, and these abnormal data were not without meaning but rather aberrant statistically. Due to the limited size of these outliers, an efficient validating experiment is yet to be implemented. In the future, more work needs to be done to validate the proposed method with a larger population and a multicenter and externally validated study.

## 5 CONCLUSION

In summary, this study validates the efficacy of the proposed DL‐based method for significantly improving the quality of lymphoma PET with a two‐fold acceleration. The SAU2Net model, employing simulated 1/2‐dose PET acquisition, consistently produced PET scans of standard quality. The results affirm the potential of the proposed method for improving clinical efficiency without compromising diagnostic accuracy Further advancements in lymphoma PET imaging and its potential integration into routine clinical practice is warranted.

## AUTHOR CONTRIBUTIONS

Author XL collected the data, conducted the clinical evaluation and write the manuscript; Author BP developed the algorithm, processed data and write the manuscript; Author CC conducted the clinical evaluation; Author DY collected the data; Author ZP revised the manuscript; Author NG lead the development of proposed algorithm; Author FL lead the clinical evaluation.

## CONFLICT OF INTEREST STATEMENT

Author BP, ZP work for RadioDynamic Healthcare. Author NG is a stock share holder of RadioDynamic Healthcare.

## ETHICS STATEMENT

Ethics approval and consent to participate: IRB approval from Beijing Hospital. All patients signed informed consent.

## CONSENT FOR PUBLICATION

All authors agree to publish.

## Data Availability

The datasets generated and analyzed during the current study are not publicly available but are available from the corresponding author on reasonable request.
